# Pharmacokinetics and safety issues of an accidental overdose of 2,000,000 IU of vitamin D_3_ in two nursing home patients: a case report

**DOI:** 10.1186/2050-6511-15-57

**Published:** 2014-09-30

**Authors:** Jody van den Ouweland, Hanneke Fleuren, Miranda Drabbe, Hans Vollaard

**Affiliations:** 1 Department of Clinical Chemistry, Canisius Wilhelmina Hospital, Weg door Jonkerbos 100, 6532 SZ Nijmegen, The Netherlands; 2 Department of Clinical Pharmacy, Canisius-Wilhelmina Hospital, Nijmegen, The Netherlands; 3 Nursing Home Waelwick, Ewijk Beuningen, The Netherlands

**Keywords:** Vitamin D, Intoxication, Single high dose

## Abstract

**Background:**

Administration of intermittent high doses of vitamin D_3_ is increasingly used as a strategy for rapid normalization of low 25-hydroxyvitamin D (25(OH)D) blood concentrations in patients with vitamin D deficiency. Here, we describe the pharmacokinetics of an accidental single oral overdose of 2,000,000 IU of vitamin D_3_ in two elderly nursing home patients and discuss safety issues.

**Case presentation:**

Two patients, a Caucasian 90-year old man and a 95-year old woman, were monitored from 1 h up to 3 months after intake for clinical as well as biochemical signs of vitamin D intoxication. Blood vitamin D_3_ concentrations showed a prompt increase with the highest peak area already hours after the dose, followed by a rapid decrease to undetectable levels after day 14. Peak blood 25(OH)D_3_ concentrations were observed 8 days after intake (527 and 422 nmol/L, respectively (ref: 50–200 nmol/L)). Remarkably, plasma calcium levels increased only slightly up to 2.68 and 2.73 mmol/L, respectively (ref: 2.20–2.65 mmol/L) between 1 and 14 days after intake, whereas phosphate and creatinine levels remained within the reference range. No adverse clinical symptoms were noted.

**Conclusion:**

A single massive oral dose of 2,000,000 IU of vitamin D_3_ does not cause clinically apparent toxicity requiring hospitalization, with only slightly elevated plasma calcium levels in the first 2 weeks. Toxicity in the long term cannot be excluded as annual doses of 500,000 IU of vitamin D_3_ for several years have shown an increase in the risk of fractures. This means that plasma calcium levels may not be a sensitive measure of vitamin D toxicity in the long term in the case of a single high overdose. To prevent a similar error in the future, the use of multiple-dose bottles need to be replaced by smaller single-unit dose formulations.

## Background

Vitamin D deficiency is a highly prevalent condition, present in approximately 30–50% of the general population [1], and may be as high as 100% in institutionalized elderly patients [2,3]. Vitamin D supplementation is increasingly advised, but compliance with daily dosing regimens is low [4]. Because serum 25-hydroxyvitamin D_3_ (25(OH)D_3_), the functional indicator of vitamin D status, has a long half-life of about 2 months [5], there is great interest in intermittent dosing for patient convenience and long-term adherence. Administration of 100,000 IU of cholecalciferol (vitamin D_3_) every 4 months for 5 years showed a 22% reduction in the risk of osteoporotic fractures [6], similar to studies using a daily dose of 800 IU [7]. On the contrary, rapid increases in serum 25(OH)D from intermittent high doses of 500,000 IU of vitamin D_3_ once a year for 3–5 years in older women, have been shown to increase the risk of falls and fractures by 26% compared with the use of a placebo [8]. In clinical studies using high-dose regimens of up to 600,000 IU of vitamin D_3_, there has been surprisingly little concern for vitamin D toxicity. Plasma calcium and urinary calcium excretion both are recognized markers of vitamin D toxicity [5]. In the few clinical studies that have analyzed plasma calcium levels and/or urinary calcium excretion within a month after dosing, calcium levels were found to be unchanged or only slightly elevated within the reference range following a loading dose of 300,000 IU [9], 500,000 IU [10], 540,000 IU [11], or 600,000 IU [12,13].

Here, we describe the pharmacokinetics of an accidental overdosing by 2,000,000 IU vitamin D_3_ in two elderly nursing home patients and discuss safety issues from single massive oral doses of vitamin D_3_.

## Case presentation

**Figure 1:**
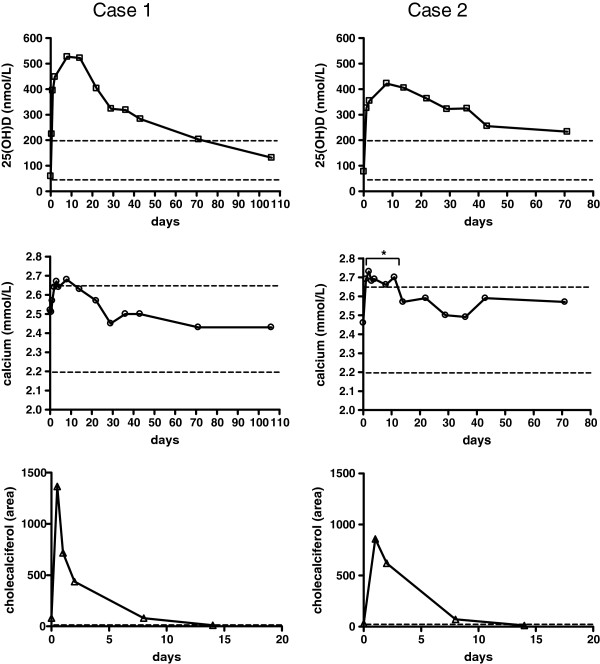
**Time course of plasma calcidiol and calcium concentrations, as well as semi-quantitative measurements of plasma vitamin D**_**3 **_**(cholecalciferol), after a single oral dose of 2,000,000 IU of vitamin D to two nursing home residents.** The asterisk indicates the individual calcium measurements that show a biologically significant difference from baseline concentrations. Note the time-scale differences between the vitamin D_3_ (cholecalciferol) and the upper plots.

In Waelwick, a nursing home near Nijmegen, all residents receive osteoporosis prophylaxis with oral vitamin D_3_ doses of 100,000 IU three times a year as a lyophilized solution (2 mL vitamin D_3_ aquosum FNA, 50,000 IU/mL). Two nursing home patients each received an accidental single overdose of 2,000,000 IU vitamin D from a whole bottle of a concentrated vitamin D_3_ solution (40 mL vitamin D_3_ aquosum FNA 50,000 IU/mL). The pharmacy department was surprised by the order for 25 new bottles immediately after the dose, as the two 40-mL bottles would have lasted for half of a year of treatment for the patients at the ward. It then became clear that an overdose had been given. For safety reasons, we immediately started biochemical and clinical monitoring of both patients. Plasma calcium, phosphate, creatinine, and 25(OH)D levels were measured from 1 h after dosing up to 106 days (case 1) and 71 days (case 2) (Figure 1, Table 1). Vitamin D_3_ was measured at time points 1 h, 0.5, 1, 2, 8, and 14 days after dosing (Figure 1). Because of the nature of this study, baseline concentrations (prior to dosing) were lacking. Unfortunately, we lacked the opportunity for measuring 1,25-dihydroxyvitamin D and 24,25-dihydroxyvitamin D (because of limited availability of plasma material), parathyroid hormone (PTH) and bone turnover markers (lack of the appropriate blood containers or pre-analytical conditions), and urinary calcium excretion (lack of urine collection).

**Table 1:** Biochemical values from single high-dose vitamin D3 administration in two cases

		**Reference range**	**1 h**	**5.5 h**	**1d**	**2d**	**3d**	**4d**	**8d**	**11d**	**14d**	**22d**	**29d**	**36d**	**43d**	**60d**	**71d**	**106d**
Case 1	Calcidiol (nmol/L)	50-200	59	**224**	**395**	**448**			**527**		**522**	**403**	**323**	**318**	**283**		**203**	131
	Calcium (mmol/L)	2.20-2.65	2.52	2.51	2.57	2.64	**2.67**	2.64	**2.68**		2.63	2.57	2.45	2.5	2.5		2.43	2.43
	Phosphate (mmol/L)	0.80-1.40	1.08	1.09	1.05	1.30	1.13	1.14	1.37		1.32	1.01	1.00	0.98	0.97			0.96
	Creatinine (μmol/L)	60-110	64	65	66	68	69	73	69		77	75	71	69	63	66	75	65
Case 2	Calcidiol (nmol/L)	50-200	77		**326**	**354**			**422**		**405**	**363**	**322**	**324**	**255**		**233**	
	Calcium (mmol/L)	2.20-2.65	2.46		**2.69**	**2.73**	**2.68**	**2.69**	**2.66**	**2.70**	2.57	2.59	2.50	2.49	2.59		2.57	
	Phosphate (mmol/L)	0.80-1.40	0.91		1.20	1.06	1.12	1.05	1.13	1.02	1.07	0.82	0.82	0.89	0.87		**0.79**	
	Creatinine (μmol/L)	50-90	**45**		51	**44**	55	54	52	52	**49**	52	**41**	50	54		**45**	

Values outside the reference range are indicated in bold. h, hours; d, days. Conversion factors: calcidiol (mg/dL = 0.4 × mmol/L), calcium (mg/dL = 4 × mmol/L), phosphate (mg/dL = 3.1 × mmol/L), creatinine (mg/dL = 0.01 × μmol/L).

Measurements of plasma 25(OH)D concentrations, as well as semi-quantitative measurement of vitamin D_3_ (monitoring peak area), were performed using liquid chromatography-tandem mass spectrometry as previously described [14]. Plasma calcium, phosphate, and creatinine levels were measured on a routine clinical chemistry analyzer (Modular; Roche Diagnostics, Mannheim, Germany). Changes of biological significance were calculated from reference change (RCV, at a 95% confidence interval) according to the formula RCV = Z × 2^1/2^$({\mathrm{CV}}_{a}^{2}+{\mathrm{CV}}_{a}^{2})$^1/2^[15], where Z = 1.96 (i.e., the Z-score for 95% confidence), CV_a_ = analytical variation, and CV_i_ = intra-individual variation. For calcium, CV_a_ and CV_i_ were 1.5% and 1.9%, respectively [16].

Case 1 was a 90-year-old man of Caucasian origin, with normal kidney function (estimated glomerular filtration rate by Modification of Diet in Renal Disease (MDRD) equation >60 mL/min), and an absence of significant pathology. He did not report any clinical signs or symptoms of vitamin D toxicity. His plasma calcium was 2.52 mmol/L 1 h after dosing and showed a non-significant increase from day 1 to day 8, with a maximum of 2.68 mmol/L at day 8. After 8 days, plasma calcium levels fell to within the reference range (2.2–2.65 mmol/L) (Figure 1). At first measurement, 1 h after gift, vitamin D3 was already detected. The vitamin D_3_ peak area showed a prompt increase with the highest peak area already at the second measurement, 5.5 h after dosing, followed by a rapid decrease to undetectable levels at day 14. Plasma 25(OH)D_3_ concentrations also were already markedly increased 5.5 h after dosing, but rose more slowly than vitamin D_3_, reaching a maximum of 527 nmol/L at day 8. Over the following 3-month period, 25(OH)D_3_ concentrations decreased by 50% over an approximately 50-day period. At the end of the 106-day follow-up period, the patient’s 25(OH)D_3_ concentration was 113 nmol/L and fell within the laboratory reference range (50–200 nmol/L). Plasma phosphate and creatinine levels showed a modest increase within the reference range (Table 1).

Case 2 relates to a 95-year-old woman of Caucasian origin, with normal kidney function (MDRD >60 mL/min) and an absence of significant pathology. She did not report any clinical signs or symptoms of vitamin D toxicity. Her plasma calcium was 2.46 mmol/L 1 h after dosing and showed a biologically significant increase from day 1 to day 11 with a maximum of 2.73 mmol/L at the second day. After 11 days, plasma calcium levels dropped to within the reference range of 2.2–2.65 mmol/L (Figure 1). Vitamin D_3_ peak area was highest at the first measurement opportunity, being 1-day post-dose, followed by a rapid decrease to undetectable levels after day 14. Plasma 25(OH)D_3_ concentrations also were already markedly increased 1 day after dosing, but rose more slowly than vitamin D_3_, reaching a maximum of 422 nmol/L at day 8. At the end of the 71-day follow-up period, the 25(OH)D_3_ concentration was 233 nmol/L, with an approximate 50% decrease from the highest level being still above the upper normal limit of 200 nmol/L. Plasma phosphate and creatinine levels both showed a modest increase within the reference range (Table 1).

## Discussion

Pharmacokinetic studies in human volunteers given a single large oral dose of 100,000 IU of vitamin D_3_ indicate that serum vitamin D_3_ and 25(OH)D_3_ levels peak on days 1 and 7, respectively [17,18]. The vitamin D_3_ concentrations fell rapidly, being close to baseline by day 7, with a much slower fall of the 25(OH)D_3_ concentrations. Plasma calcium levels did not rise at any time, and no subject experienced hypercalcemia at any of the measured time points [17]. The pharmacokinetic profiles of our two cases resembled those from the 100,000 IU study and emphasize the importance of measuring 25(OH)D and calcium concentrations over the first 14 days for identifying the maximum blood concentrations. The half-life of the plasma 25(OH)D levels of about 50 days in case 1 is comparable to a half-life of about 2 months as described previously [5]. The relatively normal 25(OH)D_3_ concentrations in our nursing home residents (59 nmol/L in case 1 and 77 nmol/L in case 2) at the first measurement (1 h post-dosing) can be explained as the result of previous osteoporosis prophylaxis treatments with 100,000 IU of oral vitamin D_3_. However, we cannot exclude the possibility that the plasma 25(OH)D concentrations were already increased 1 h after the high dose of vitamin D_3_, implicating that the baseline 25(OH)D values in both cases actually may have been lower.

The toxicity of vitamin D in daily administration has been studied extensively. The present view is that hypercalcemia is the hazard criterion for vitamin D [19]. Thus, vitamin D toxicity is supposed to be inseparable from hypercalcemia. Hypercalcemia does not occur with daily doses up to 40,000 IU of vitamin D_3_ and serum levels of 25(OH)D_3_ up to 500 nmol/L, well above naturally occurring levels of 25(OH)D_3_[20]. Most patients with an overdose of vitamin D_3_ are not seen by a physician until they are admitted to the emergency department with clinical signs of intoxication, such as life-threatening dehydration, after a prolonged period of oral intake of high-dose vitamin D, either from contaminated food components [21,22] or supplements with errors in manufacturing and/or labeling [23,24].

Much less is known about the toxicity of vitamin D_3_ following a single high oral dose of vitamin D_3_. In studies using 300,000 IU [9], 540,000 IU [11], and 600,000 IU [12,13] of oral vitamin D_3_, no adverse effects were noted and plasma calcium levels or urinary calcium excretion did not change, or only slightly increased for a short period of time, with all cases remaining within the physiological range. In contrast to these reassuring data, Sanders et al. [8] found a significant increase in the risk of falls (+15%) and fractures (+26%) in a randomized controlled trial with an annual dose of 500,000 IU vitamin D_3_ for 3–5 years in older women compared with placebo. Falls and fractures occurred especially within the first 3 months following the annual dose of vitamin D_3_. In that study, plasma calcium levels and urinary calcium excretion were not measured; however, bearing in mind the former data, it is unlikely that hypercalcemia occurred in the study. These unexpected findings led Rossini et al. [13,25] to investigate dose-dependent short-term effects on bone turnover markers of a single bolus of vitamin D_3_. A single dose of 600,000 IU of vitamin D_3_ increased serum C-terminal telopeptide of type 1 collagen and cross-linked N-telopeptide of type 1 collagen significantly at day 1, attained a peak increment greater than 50% at day 3, and subsequently decreased almost back to baseline values at day 90, without statistically significant changes in plasma calcium [13]. Their results indicate that the use of oral megadoses greater than 100,000 IU of vitamin D may be counterproductive and that the safety issues surrounding vitamin D dosing should not be limited to early markers of vitamin D toxicity as plasma hypercalcemia or increased urinary calcium excretion [13,25]. A possible explanation is the fact that the level of 1,25 dihydroxy-vitamin D is regulated much stronger in the endocrine system than in the autocrine system of vitamin D [5]. If the plasma level of calcidiol (the substrate of the autocrine vitamin D system) increases sharply after a loading dose that deviates too much from the maximum physiological daily dose with sunlight (20,000 IU), this might result in toxic intracellular calcitriol levels in the autocrine vitamin D system without toxic circulating calcitriol levels in the endocrine system of vitamin D and without hypercalcemia. If that is the case, we need other markers than hypercalcemia to prove vitamin D toxicity in the autocrine system, such as bone turnover markers [13]. In any case, more studies are needed to evaluate potential adverse effects of high-dose vitamin D treatment on bone metabolism in relation to fracture risk and risk of falls.

The present study displays some limitations. We lacked the opportunity for studying other relevant parameters in the risk assessment for vitamin D_3_, such as measurement of urinary calcium excretion and plasma levels of 1,25-dihydroxyvitamin D, 24,25-dihydroxyvitamin D, PTH, and bone turnover markers. Because of the nature of this study, baseline concentrations (prior to dosing) of biochemical parameters such as plasma vitamin D_3_, 25(OH)D, and calcium were lacking.

## Conclusion

In our two patients, the massive single overdose of 2,000,000 IU of vitamin D_3_ did not result in immediate clinical and biochemical toxicity requiring hospitalization, with only slightly elevated plasma calcium levels in the first 2 weeks. However, toxicity in the long term cannot be excluded as an annual dose of 500,000 IU of vitamin D_3_ for several years has shown an increased risk of fractures. This may implicate that markers other than plasma calcium measurement, such as bone turnover markers, are needed to assess the safety of high doses of vitamin D in the long term. Clearly, the safety of high-dose vitamin D supplementation warrants further study. To prevent a similar error in the future, the use of multiple-dose bottles were replaced by smaller single-unit dose formulations of 25.000 IU/mL. In general, we believe that available formulations of vitamin D_3_ should not exceed 100,000 IU, as long as the safety of high doses of vitamin D in the long term has not been established.

## Consent

Written informed consent for publication of their clinical details was obtained from the relatives of both patients. A copy of the written consent is available for review by the Editor of this journal.

## Abbreviations

25(OH)D: 25-hydroxyvitamin D; CV_a_: analytical variation; CV_i_: intra-individual variation; RCV: reference change value.

## Competing interests

The authors declare that they have no competing interests.

## Authors’ contributions

MD was the treating physician of the patients reported. JvdO carried out the biochemical measurements, evaluated the test results and drafted the manuscript. HF and HV evaluated the test results and helped to draft the manuscript. All authors read and approved the final version of the manuscript.

## Pre-publication history

The pre-publication history for this paper can be accessed here:

http://www.biomedcentral.com/2050-6511/15/57/prepub

## References

[B1] Holick MF Vitamin D: evolutionary, physiological and health perspectives Curr Drug Targets 2011 12 4 18 10.2174/138945011793591635 20795941

[B2] Lips P Vitamin D deficiency and secondary hyperparathyroidism in the elderly: consequences for bone loss and fractures and therapeutic implications Endocr Rev 2001 22 477 501 10.1210/edrv.22.4.0437 11493580

[B3] Pilz S Dobnig H Tomaschitz A Kienreich K Meinitzer A Friedl C Wagner D Piswanger-Sölkner C März W Fahrleitner-Pammer A Low 25-hydroxyvitamin D is associated with increased mortality in female nursing home residents J Clin Endocrinol Metab 2012 97 E653 E657 10.1210/jc.2011-3043 22319037

[B4] Bischoff-Ferrari HA How to select the dose of vitamin D in the management of osteoporosis Osteoporosis Int 2007 18 401 407 10.1007/s00198-006-0293-9 17151835

[B5] Vieth R Vitamin D The Pharmacology of Vitamin D Chapter 57 in Feldman, Pike and Adams 2011 3, volume II London, UK: Academic

[B6] Trivedi DP Dolf R Khaw KT Effect of four monthly oral vitamin D3 (cholecalciferol) supplementation on fractures and mortality in men and women living in the community: randomised double blind controlled trial BMJ 2003 326 469 475 10.1136/bmj.326.7387.469 12609940 PMC150177

[B7] Dawson-Hughes B Heaney RP Holick MF Lips P Meunier PJ Vieth R Estimates of optimal vitamin D status Osteoporosis Int 2005 16 713 716 10.1007/s00198-005-1867-7 15776217

[B8] Sanders KM Stuart AL Williamson EJ Simpson JA Kotowicz MA Young D Nicholson GC Annual high-dose oral vitamin D and falls and fractures in older women. A randomized controlled trial JAMA 2010 303 1815 1822 10.1001/jama.2010.594 20460620

[B9] Romagnoli E Mascia ML Cipriani C Fassino V Mazzei F D’Erasmo E Carnevale V Scillitani A Minisola S Short and long-term variations in serum calciotropic hormones after a single very large dose of ergocalciferol (vitamin D2) or cholecalciferol (vitamin D3) in the elderly J Clin Endocrinol Metab 2008 93 3015 3020 10.1210/jc.2008-0350 18492750

[B10] Bacon CJ Gamble GD Horne AM Scott MA Reid IR High-dose oral vitamin D3 supplementation in the elderly Osteoporos Int 2009 20 1407 1415 10.1007/s00198-008-0814-9 19101755

[B11] Amrein K Sourij H Wagner G Holl A Pieber TR Smolle KH Stojakovic T Schnedl C Dobnig H Short term effects of high dose oral vitamin D3 in critically ill vitamin D deficient patients: a randomized, double blind, placebo controlled pilot study Crit Care 2011 15 R104 10.1186/cc10120 21443793 PMC3219377

[B12] Cipriani C Romagnoli E Scillatani A Chiodini I Clerico R Carnevale V Mascia ML Battista C Viti R Pileri M Eller-Vainicher C Minisola S Effect of a single oral dose of 600,000 IU of cholecalciferol in serum calciotropic hormones in young subjects with vitamin D deficiency: A prospective intervention study J Clin Endocrinol Metab 2010 95 4771 4777 10.1210/jc.2010-0502 20660032

[B13] Rossini M Gatti D Viapiana O Fracassi E Idolazzi L Zanoni S Adami S Short term effects on bone turnover markers of a single high dose of oral vitamin D3 J Clin Endocrinol Metab 2012 97 E622 E626 10.1210/jc.2011-2448 22298802

[B14] van den Ouweland JM Beijers AM Demacker PN van Daal H Measurement of 25-OH-vitamin D in human serum using liquid chromatography tandem-mass spectrometry with comparison to radioimmunoassay and automated immunoassay J Chromatogr B Analyt Technol Biomed Life Sci 2010 878 1163 1168 10.1016/j.jchromb.2010.03.035 20381436

[B15] Fraser CG Biological Variation: From Principles to Practice 2001 Washington: AACC press 67 70

[B16] Ricós C Iglesias N García-Lario JV Simón M Cava F Hernández A Perich C Minchinela J Alvarez V Doménech MV Jiménez CV Biosca C Tena R Within-subject biological variation in disease: collated data and clinical consequences Ann Clin Biochem 2007 44 343 352 10.1258/000456307780945633 17594781

[B17] Ilahi M Armas LA Heaney RP Pharmacokinetics of a single, large dose of cholecalciferol Am J Clin Nutr 2008 87 688 691 18326608 10.1093/ajcn/87.3.688

[B18] Heaney RP Armas LA Shary JR Bell NH Binkley N Hollis BW 25-Hydroxylation of vitamin D3: relation to circulating vitamin D3 under various input conditions Am J Clin Nutr 2008 87 1738 1742 18541563 10.1093/ajcn/87.6.1738

[B19] Vieth R Vitamin D toxicity, policy, and science J Bone Miner Res 2007 22 Suppl 2 V64 V68 18290725 10.1359/jbmr.07s221

[B20] Vieth R Vitamin D supplementation, 25-hydroxyvitamin D concentrations and safety Am J Clin Nutr 1999 69 842 856 10232622 10.1093/ajcn/69.5.842

[B21] Vieth R Pinto TR Reen BS Wong MM Vitamin D poisoning by table sugar Lancet 2002 359 672 10.1016/S0140-6736(02)07814-5 11879864

[B22] Down PF Polak A Regan RJ A family with massive acute vitamin D intoxication Postgrad Med J 1979 55 897 902 10.1136/pgmj.55.650.897 232912 PMC2425685

[B23] Araki T Holick MF Alfonso BD Charlap E Romero CM Rizk D Newman LG Vitamin D intoxication with severe hypercalcemia due to manufacturing and labeling errors of two dietary supplements made in the United States J Clin Endocrinol Metab 2011 96 3603 3608 10.1210/jc.2011-1443 21917864

[B24] Lowe H Cusano NE Binkley N Blaner WS Bilezikian JP Vitamin D toxicity due to a commonly available “over the counter” remedy from the Dominican Republic J Clin Endocrinol Metab 2011 96 291 295 10.1210/jc.2010-1999 21123442

[B25] Rossini M Adami S Viapiana O Fracassi E Idolazzi L Povine MR Gatti D Dose-dependent short-term effects of single high doses of oral vitamin D3 on bone turnover markers Calcif Tissue Int 2012 91 365 369 10.1007/s00223-012-9637-y 23052222

